# Study on the role and pharmacology of cuproptosis in gastric cancer

**DOI:** 10.3389/fonc.2023.1145446

**Published:** 2023-03-17

**Authors:** Lin Jiang, Junzuo Liao, Yunwei Han

**Affiliations:** ^1^ Department of Oncology, The First Affiliated Hospital of Jinan University, Guangzhou, China; ^2^ Department of Oncology, The Second Affiliated Hospital of North Sichuan Medical College, Nanchong, Sichuan, China; ^3^ Department of General Surgery, Affiliated Hospital of North Sichuan Medical College, Nanchong, Sichuan, China; ^4^ Department of Oncology, The Affiliated Hospital of Southwest Medical University, Luzhou, Sichuan, China

**Keywords:** gastric cancer, cuproptosis, Diagnostic model, molecular docking, cancer

## Abstract

**Objective:**

Gastric cancer has a poor prognosis and high mortality. Cuproptosis, a novel programmed cell death, is rarely studied in gastric cancer. Studying the mechanism of cuproptosis in gastric cancer is conducive to the development of new drugs, improving the prognosis of patients and reducing the burden of disease.

**Methods:**

The TCGA database was used to obtain transcriptome data from gastric cancer tissues and adjacent tissues. GSE66229 was used for external verification. Overlapping genes were obtained by crossing the genes obtained by differential analysis with those related to copper death. Eight characteristic genes were obtained by three dimensionality reduction methods: lasso, SVM, and random forest. ROC and nomogram were used to estimate the diagnostic efficacy of characteristic genes. The CIBERSORT method was used to assess immune infiltration. ConsensusClusterPlus was used for subtype classification. Discovery Studio software conducts molecular docking between drugs and target proteins.

**Results:**

We have established the early diagnosis model of eight characteristic genes (ENTPD3, PDZD4, CNN1, GTPBP4, FPGS, UTP25, CENPW, and FAM111A) for gastric cancer. The results are validated by internal and external data, and the predictive power is good. The subtype classification and immune type analysis of gastric cancer samples were performed based on the consensus clustering method. We identified C2 as an immune subtype and C1 as a non-immune subtype. Small molecule drug targeting based on genes associated with cuproptosis predicts potential therapeutics for gastric cancer. Molecular docking revealed multiple forces between Dasatinib and CNN1.

**Conclusion:**

The candidate drug Dasatinib may be effective in treating gastric cancer by affecting the expression of the cuproptosis signature gene.

## 1 Introduction

Gastric cancer is one of the most common malignant tumors in the world, with the third highest mortality rate from cancer. In 2020, the number of new cases of stomach cancer in the world exceeded 1 million, and 769,000 people died from stomach cancer (1). East Asia is the concentrated area of the increasing gastric cancer cases in the world, and our country is the increasing main country in East Asia (2). The National Cancer Center reports that gastric cancer ranks second place in the incidence rate of malignant tumors and third in the mortality rate, posing a serious threat to the health of residents (3). The development of gastric cancer is a complex evolutionary process involving many factors and genes (4). Helicobacter pylori infection is the most important risk factor for gastric cancer. In addition, excessive consumption of preserved foods, alcohol consumption, and smoking are also risk factors for an increased risk of gastric cancer (5, 6). The molecular mechanism of gastric cancer is not fully understood. Current studies suggest that gastric mucosal epithelial cells undergo gene mutations under the influence of a number of complex factors, which then activate proto-oncogenes or silence tumor suppressor genes, thereby disrupting the balance between cell proliferation and apoptosis, and ultimately leading to the development of gastric cancer (7, 8). According to Lauren’s classification, gastric cancer is mainly an intestinal type (9). The occurrence of intestinal gastric cancer is a multi-step cascade reaction: non-atrophic gastritis-multifocal atrophic gastritis with metaplasia-intestinal metaplasia-intraepithelial neoplasia-early gastric cancer-invasive advanced gastric cancer (10). Most of the previous studies have focused on advanced gastric cancer, while there are relatively few studies on abnormal molecular expression in early gastric cancer. The treatment and prognosis of gastric cancer are closely related to the timing of diagnosis. The 5-year survival rate of early gastric cancer patients after eradication is more than 90%, while the 5-year survival rate of advanced gastric cancer patients after eradication is less than 30% (11). In recent years, with the gradual enhancement of people’s health awareness and the continuous progress of medical technology, the diagnosis rate of early gastric cancer has been greatly improved. The molecular mechanism of early gastric cancer is a hot topic in translational medicine in recent years.

With the rapid development of life sciences, studies on genomics, transcriptomics, proteomics, and metabolomics are emerging in an endless stream, which making it possible to analyze the molecular map of different stages of cancer transformation of gastric cancer from multiple dimensions, facilitating the monitoring of the occurrence, metastasis and drug resistance of gastric cancer. Futawatari et al. found that KK-LC-1 was abnormally highly expressed in early gastric cancer tissues, which could be used as a tumor marker for the diagnosis of early gastric cancer (12). Through genome-wide expression profiling microarray analysis, Zhang et al. found that the expression levels of GRIN2D and BRCAl in early gastric cancer and intraepithelial neoplasia were much higher than those in paired normal gastric mucosa, while the expression levels of BCL2L11, RET, and ALB were lower (13). Therefore, if the genes that regulate the changes in the progression of early gastric cancer can be screened and the specific mechanism of action can be clarified, it will be of great importance in the search for new targets of gastric cancer from the source.

Copper is an essential nutrient whose REDOX properties make it both beneficial and toxic to cells (14). Due to the high demand for copper as a metallic nutrient in tumor growth and metastasis, copper-related diagnostic methods are well suited for tumors (14). The traditional view of copper as merely a cofactor of active site metabolism has been challenged. A recent study has shown that intracellular copper induces a novel form of regulatory cell death (RCD), which differs from traditional cell death and has been termed “cuproptosis” (15). Cuproptosis is a type of programmed cell death that is distinct from apoptosis and may offer provide new hope for the treatment of gastric cancer. Although scientists have identified a number of genes and proteins that regulate cuproptosis, including FDX1, LIAS, DLAT, and CNN1, among others (15). However, the mechanism of action of these cuproptosis-related genes (CRGs) in gastric cancer remains unclear. Little is also known about the role of CRGs in diagnosis and the tumor microenvironment. Recent studies have reported that cuproptosis is closely related to cancer progression (15). There is increasing evidence that cuproptosis-associated long non-coding RNAs can be used as biomarkers for the prognosis of gastric cancer (16–18). However, the study on cuproptosis-related genes in early diagnosis and treatment of gastric cancer has not been reported. Therefore, in-depth understanding of the characteristics of TME immune cell infiltration mediated by many CRGs will help researchers better understand the potential mechanism of gastric cancer, predict the immune treatment response, and develop new safe and efficient targeted drugs.

## 2 Materials and methods

### 2.1 Microarray data set and difference analysis

Microarray datasets from gastric cancer patients and adjacent tissues were obtained from the TCGA database. The limma package in R was then used to identify and standardize differentially expressed genes (DEGs) by comparing the gene expression levels of gastric cancer patients and adjacent tissues (19). *P* < 0.05 and | logFC | > 1 were used to define the standard of DEG. The ACRG (Asian Cancer Research Group) dataset GSE66229 was used for external validation.

### 2.2 Analysis of cuproptosis and immune-related genes

From a genome-wide CRISPR-Cas9 dysfunction test reported in the previous literature (15), a total of 347 potential copper-associated genes were identified (FDR<0.05). The list of 1793 immune-related genes were obtained from the Immunology Database and Analysis Portal (ImmPort; https://www.immport.org/home).

### 2.3 Functional annotation and pathway enrichment analysis

ClusterProfiler packages are used for functional analysis of biological functions, including Gene Ontology (GO) and the Kyoto Encyclopedia of Genes and Genomes (KEGG). *P* values are adjusted using the Benjamini-Hochberg method or FDR for multiple testing corrections. The threshold is set to FDR<0.05. The GO category includes biological processes (BP), molecular functions (MF), and cellular components (CC). GENEMANIA (http://genemania.org/search/) was used to build a gene-interaction network for DEGs to evaluate the function of these genes.

### 2.4 Selection of characteristic genes

Three machine learning algorithms, LASSO, Random Forest, and SVM-RFE, were used to screen the trait genes. LASSO is a dimensionality reduction method that has been shown to be superior to regression analysis in evaluating high-dimensional data. The LASSO analysis was performed using the steering/penalty parameters with 10x cross-validation *via* the glmnet package. Recursive Feature Elimination (RFE) of the Random Forest algorithm is a supervised machine learning method for sequencing genes associated with atherosclerotic plaque progression and immunity. The predicted performance was estimated by ten-fold cross-validation. SVM-RFE is superior to linear discriminant analysis (LDA) and means square error (MSE) methods in selecting correlation features and removing redundant features. SVM-RFE was applied to feature selection by ten-fold cross-validation. The receiver operating characteristic (ROC) curve and area under the curve (AUC) were used to estimate the diagnostic effectiveness.

### 2.5 Establishment of a line graph

The rms package was used to incorporate characteristic genes to create a column map. Calibration curves are used to assess the accuracy of a column plot. The clinical practicability of the line map was assessed by decision curve analysis.

### 2.6 Estimation of immune cell infiltration in gastric cancer

The CIBERSORT algorithm was used to estimate the proportion of immune cell infiltration in gastric cancer samples. Estimates of immune cell infiltration with *P*<0.05 were used for further analysis.

### 2.7 Consensus cluster analysis

Based on the expression profile of gastric cancer and cuproptosis-associated genes, the number of unsupervised categories in gastric cancer was quantitatively estimated by the ConsensusClusterPlus software package (50 iterations and 80% resampling rate) using the consensus clustering method (20). The consensus matrix graph, consensus cumulative distribution function (CDF) graph, the relative change in area under the CDF curve, and tracking graph were used to find the optimal clustering number. Principal component analysis (PCA) was used to define differences in the expression of gastric cancer and cuproptosis-related genes between the two subtypes. The PCA plot was generated using the ggplot2 package.

### 2.8 Small molecule drug prediction

We used the three characteristic genes selected by a gene-set enrichment network tool Enrichr based on the Drug Characterization Database (DSigDB) to predict potential drugs. DSigDB is a free Web-based repository of information on GSEA drugs and their target genes. DSigDB currently contains a total of 22,527 genomes, including 17,389 drugs and 19,531 genes. *P*<0.05 was used as the statistical criterion to identify drugs that were significantly associated with target genes.

### 2.9 Molecular docking

For molecular docking, Dasatinib was selected as the receptor target in this study. The 3D crystal structures of these receptors were downloaded from the RCSB Protein database (http://www.rscb.org/pdb/). PubChem ligand from the national library of medicine (https://pubchem.ncbi.nlm.nih) to download and save the data file format for the space (SDF). The Automatic Docking Tool version 1.5.6 was used to prepare protein ligand complexes for docking and for 2D and 3D visualization of protein ligand complexes, operated using the Discovery Studio Visualization tool 2016.

## 3 Results

### 3.1 Microarray data sets and difference analysis

**Figure 1:**
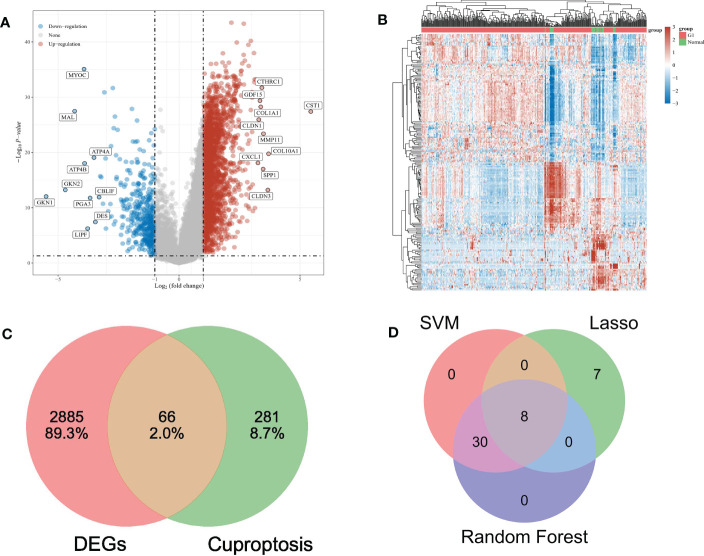
**(A)** The volcano map depicts the RNA expression levels of differential genes between gastric and paracancer tissues. **(B)** Heat maps showing differentially expressed genes between the above groups. **(C)** VENN diagrams show the intersection of differential genes and cuproptosis-related genes. **(D)** VENN diagram shows the intersection of three feature genes screened by machine learning.

The mRNA expression profile of gastric cancer was retrieved based on the TCGA database, and 375 cancer tissues and 32 para-carcinoma tissues were obtained. The limma package in R was used for the identification and standardization of differentially expressed genes (DEG). The threshold was set as *P* < 0.05 and | logFC | > 1, and 2951 differentially expressed genes were obtained. There were 2,532 up-regulated genes and 419 down-regulated genes. The DEGs data is visualized as A volcano map (**Figure 1A**) and the first 50 DEGs are shown in a heat map (**Figure 1B**). The basic information is in the supplementary documents.

### 3.2 Analysis of cuproptosis-related genes

347 cuproptosis genes were collected according to relevant literature. Intersecting with DEGs, 66 overlapping genes (OG) were obtained (**Figure 1C**).

### 3.3 GO term and KEGG pathway enrichment analysis of OG

**Figure 2:**
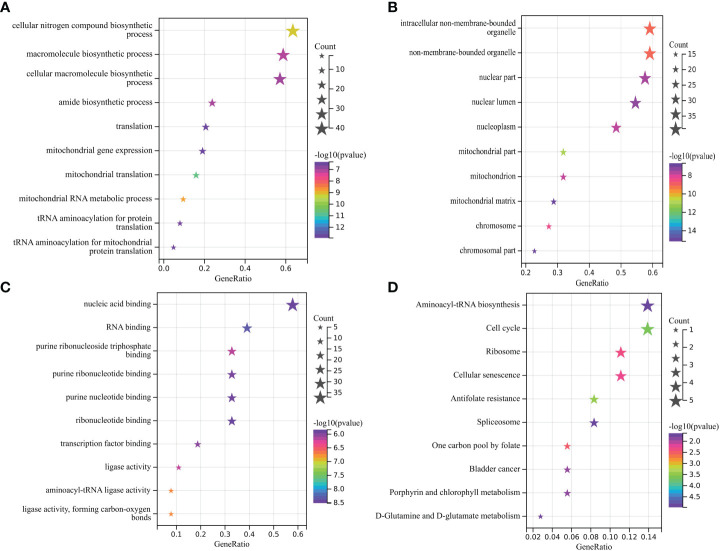
**(A-C)** Main BP, CC, and MF of overlapping gene enrichment. **(D)** Major KEGG pathways for the enrichment of the above overlapping genes.

GO analysis shows that the biological process (BP) of OG mainly focuses on the cellular nitrogen compound biological process, macroporous biological process, and cellular macroporous biological process (**Figure 2A**). The main cell components (CC) include intelligent non membrane-bound organelle, on-membrane-bounded organelle, and nuclear part (**Figure 2B**). Molecular function (MF) includes nuclear acid binding, RNA binding, and purine ribonuclease triphosphate binding (**Figure 2C**). Genes are mainly involved in the KEGG pathway of Aminoacyl tRNA biosynthesis, Cell cycle and Ribome (**Figure 2D**).

### 3.4 Analysis of protein interaction network of OGs

**Figure 3:**
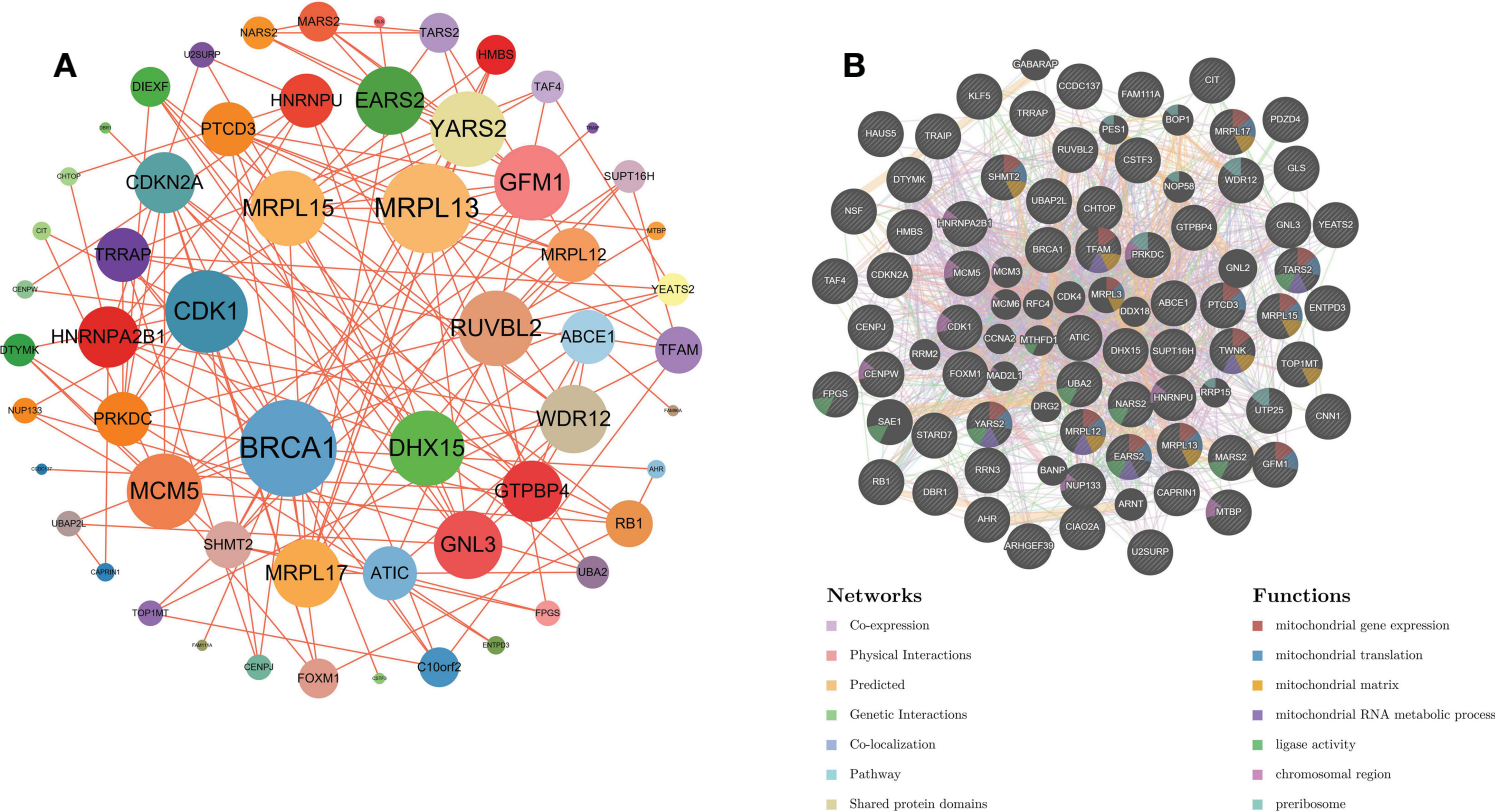
**(A)** PPI network of overlapping genes. **(B)** The GeneMANIA database was used to analyze the gene-gene interaction network of OG. Each node represents a gene. The node color represents the possible function of the corresponding gene.

Based on the string website, we obtained the protein interaction network of the OG gene set. The software Cytoscape was used to present the results. The larger the circular area of the gene, the higher the degree score and the greater the importance. This shows that the element gene of the central circle is very important (**Figure 3A**). In addition, based on GeneMANIA’s functional annotation model, a co-expression network was established to describe the genetic interaction of 66 OGs and their co-expressed genes (**Figure 3B**). Multiple attributes based on relationship (57.28% co-expression), (17.78% physical interaction), (10.91% prediction), (9.27% genetic interaction), (4.55% co-location). Of the 66 OGs, 13 were highly correlated with mitochondrial gene expression (adj. *P*=3.87E-9), and 11 were highly correlated with mitochondrial translation reaction (adj. *P*=5.28E-8) (**Figure 3B**).

### 3.5 Select characteristic genes through LASSO, random forest, and SVM-RFE algorithm

**Figure 4:**
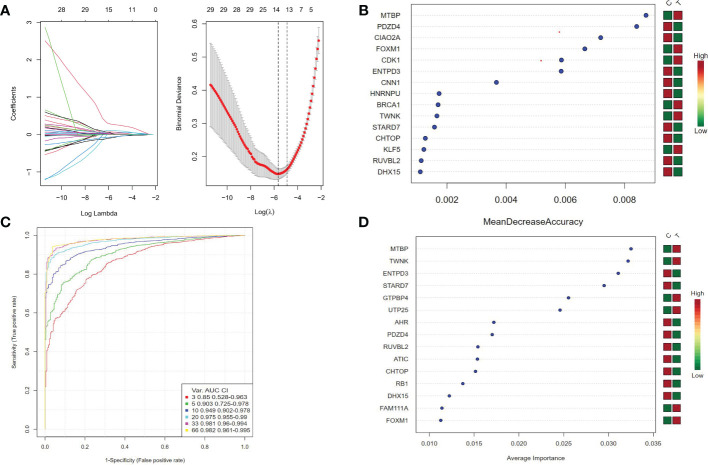
**(A)** Ten cross-validation of tuning parameter selection in the LASSO model. Each curve corresponds to a gene. LASSO coefficient analysis. The vertical solid line represents the partial likelihood deviation SE. The vertical dotted line is drawn at the best lambda. **(B)** Random forest algorithm for feature selection. **(C, D)** SVM-RFE algorithm for feature selection.

Three algorithms are used to select feature genes. For the LASSO algorithm, after ten cross-validation, we selected the minimum standard for constructing LASSO classifier, because the accuracy of comparison is higher, and 15 characteristic genes were identified (**Figure 4A**). For the random forest algorithm, 38 characteristic genes were identified (**Figure 4B**). For the SVM-RFE algorithm, 38 characteristic genes were also identified (**Figures 4C, D**). After cross-validation, eight characteristic genes (ENTPD3, PDZD4, CNN1, GTPBP4, FPGS, UTP25, CENPW, and FAM111A) shared by LASSO, Random Forest, and SVM-RFE algorithm were finally determined (**Figure 1D**).

### 3.6 Diagnostic efficacy and external validation of characteristic genes in predicting gastric cancer

**Figure 5:**
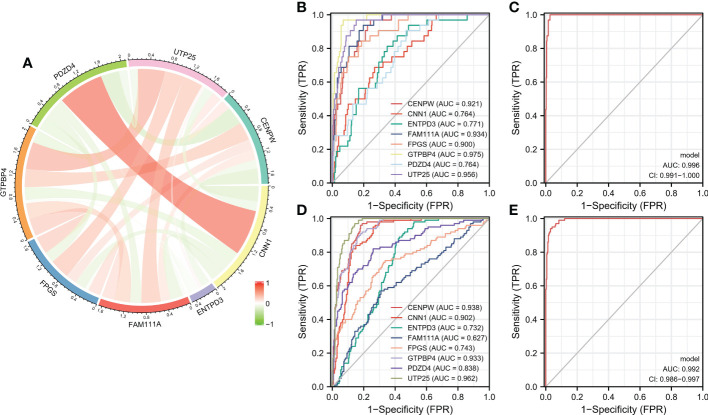
**(A)** Circle chart of characteristic gene correlation analysis. **(B, C)** ROC curve for estimating the diagnostic performance of characteristic genes. **(D, E)** ROC curve of externally verified characteristic genes.

Correlation analysis shows that there is a strong correlation between the eight characteristic genes (**Figure 5A**). When the eight characteristic genes (ENTPD3, PDZD4, CNN1, GTPBP4, FPGS, UTP25, CENPW, and FAM111A) are all fitted into one variable, the AUC of the ROC curve is 0.996, indicating a good diagnostic efficiency for gastric cancer (**Figures 5B, C**). We also estimated the diagnostic performance of each characteristic gene in predicting gastric cancer in the GSE126307 cohort. The AUC values of area under the ROC curve of 8 characteristic genes are very good, which proves that these characteristic genes can estimate the occurrence of gastric cancer. The expression of the characteristic genes was verified in the external data set. In the GSE66,229 dataset, the AUC value of the area under the ROC curve of eight characteristic genes (ENTPD3, PDZD4, CNN1, GTPBP4, FPGS, UTP25, CENPW, and FAM111A) is also high. When fitting together, the AUC of the ROC curve is 0.992, which shows that they can distinguish gastric cancer from healthy controls (**Figures 5D, E**). Therefore, the signature genes have excellent diagnostic performance in predicting the occurrence of gastric cancer.

### 3.7 Establishment of characteristic gene nomogram

**Figure 6:**
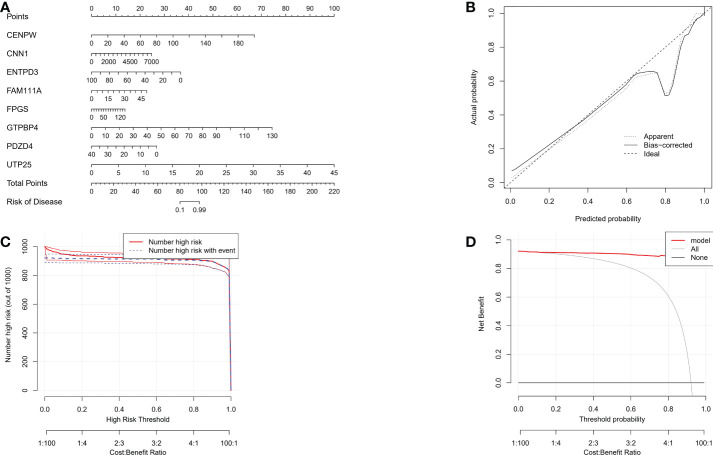
**(A)** The nomogram of integrated characteristic genes was established to predict the occurrence of gastric cancer. In the nomogram, each variable corresponds to a score, and the total score can be calculated by adding the scores of all variables. **(B)** Prediction accuracy of calibration curve estimation nomogram. **(C)** Risk comparison curve of nomograph. **(D)** Clinical decision curve of the nomogram.

In the nomogram, each characteristic gene corresponds to a score, and the total score is obtained by adding the scores of all the characteristic genes. The total score corresponds to different risks of gastric cancer (**Figure 6A**). The calibration curve, risk comparison, and clinical decision curve show that a nomogram can accurately predict the occurrence of gastric cancer (**Figures 6B–D**).

### 3.8 Analysis of immune cell infiltration and correlation in gastric cancer

**Figure 7:**
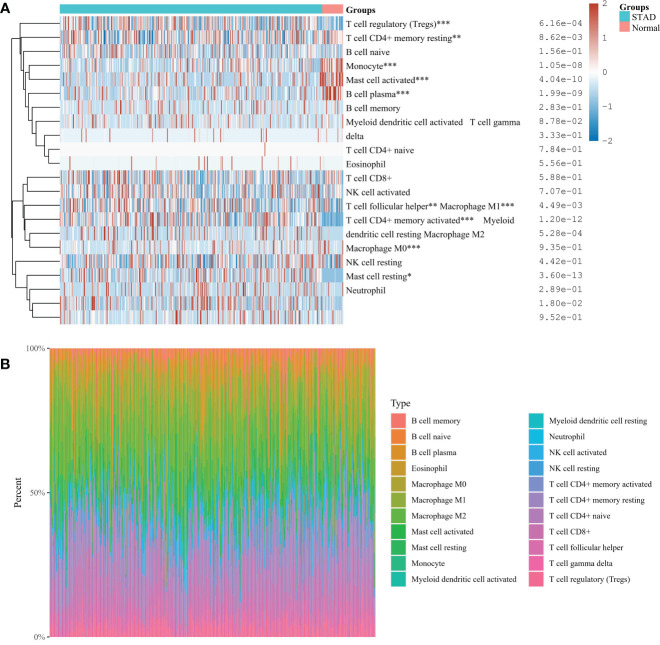
**(A)** Immunocyte score heat map *P< 0.05, **P < 0.01, ***P < 0.001. **(B)** The percentage abundance of tumor-infiltrating immune cells in each sample. Different colors represent different types of immune cells, the abscissa represents the sample, and the ordinate represents the percentage of immune cells in a single sample.

**Figure 8:**
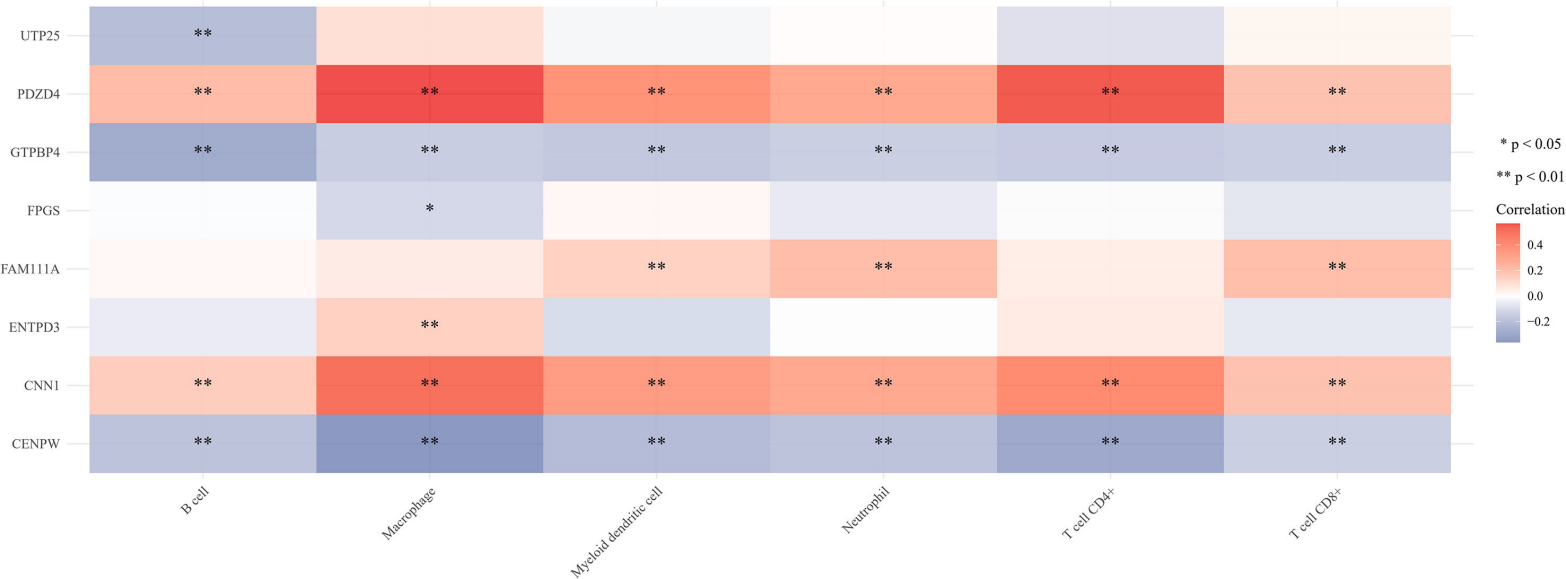
Correlation between eight characteristic genes and immune cells. *P<0.05, **P<0.01.

The proportion of immune cells in gastric cancer tissue samples and adjacent tissues is different. Compared with adjacent tissues, the proportion of B cell plasma, T cell CD4+memory resetting, Monocyte, and mast cell activated in the cancer group is relatively high, while the proportion of T cell CD4+memory activated, T cell follicular helper, T cell regulatory (Tregs), Macrophage M0, Macrophage M1, and mast cell resetting is relatively low (**Figures 7A, B**). Correlation analysis showed that there was a strong correlation between the eight characteristic genes and immune cells. It shows that the cuproptosis gene may influence the degree of immune invasion of gastric cancer (**Figure 8**).

### 3.9 Construction of two subtypes of copper dead gastric cancer based on gastric cancer and cuproptosis-related genes

**Figure 9:**
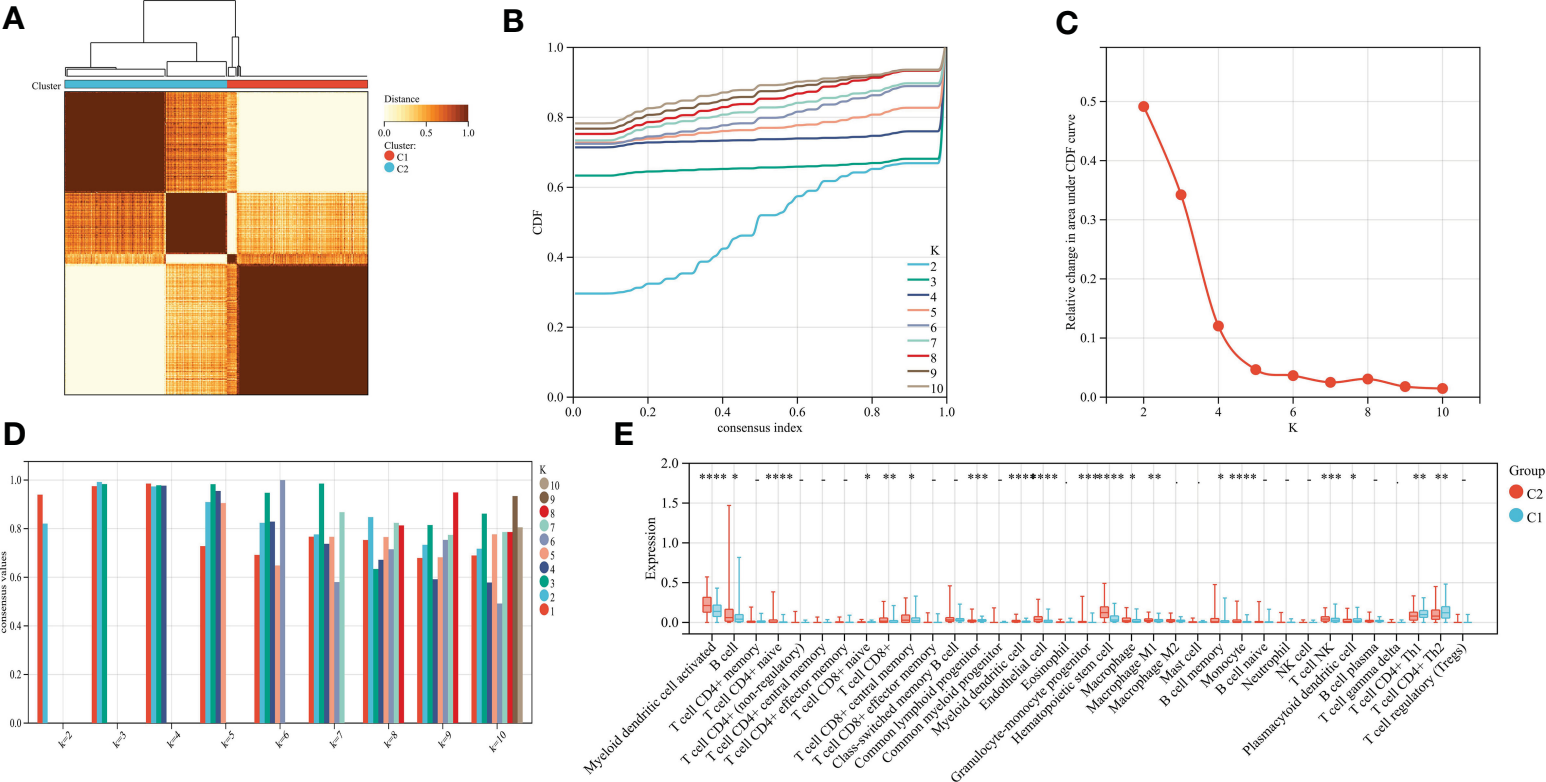
**(A)** Consensus matrix heat map when k=2. **(B)** Consensus CDF when k=2-9. **(C)** The relative change of area under the CDF curve. **(D)** When k=2-9, the tracking chart of sample classification is displayed. **(E)** Histogram of distribution of immune cells in subtype grouping.

Using the consensus clustering method, gastric cancer was clustered according to the expression profiles of 66 gastric cancer and cuproptosis-related genes. The optimal number of subtypes is 2, as determined by the consensus matrix, the CDF chart, the relative change of area under the CDF curve, and tracking chart (**Figures 9A–D**). We noticed that most immune-related genes were significantly up-regulated in subtype C2 compared with subtype C1 (**Figure 9E**). We identified C2 as an immune subtype and C1 as a nonimmune subtype.

### 3.10 Prediction and molecular docking of targeted drugs for gastric cancer

**Figure 10:**
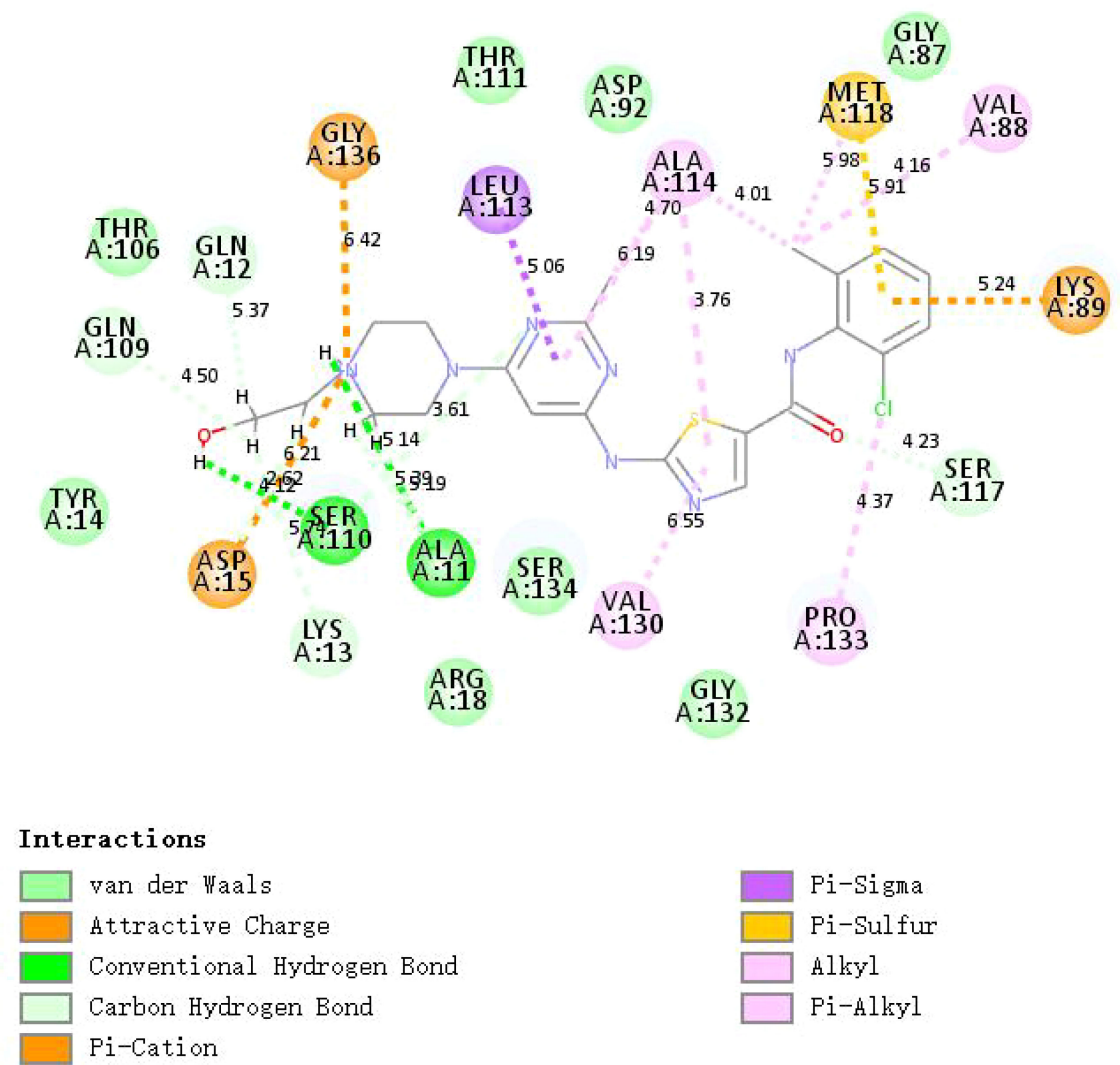
The 2D image shows the docking complex of Dashatinib and CNN1.

Further, we screened the candidate drugs that may be used to treat gastric cancer. We consider the eight selected characteristic genes as drug targets and use the online network tool Enrichr based on DSigDB for drug target enrichment analysis. The results show that the top ten drugs may be potential drugs for the treatment of gastric cancer patients (**Table 1**). To verify the above results, we performed molecular docking between small molecule drugs and target genes, and the results showed that there are multiple forces between Dasatinib and CNN1. For example, multiple forces including hydrogen bonds can be formed (**Figure 10**). The above results indicate that candidate drugs may achieve the effect of treating gastric cancer by influencing the expression of characteristic genes.

**Table 1:** Complete basic information was obtained from 261 follow-up data.

Characteristics	Cases
Gender	
Male	181
Female	80
Age at surgery	
<58	121
≥58	140
Tumor size	
<5 cm	131
≥5 cm	130
Histological type	
Diffuse	75
Intestinal	186
T classification	
T1–2	39
T3–4	222
TNM stage	
I + II	48
III + IV	213
Lymph node metastasis	
Present	216
Absent	45

## 4 Discussion

We established a diagnostic model of cuproptosis for gastric cancer based on machine learning and other methods, predicted potential therapeutic drugs based on cuproptosis-related genes, and finally performed a virtual combination of molecular docking space structure for therapeutic drugs. The mRNA expression profiles of gastric cancer were obtained from the TCGA database, and 375 cases of cancer tissues and 32 cases of para-carcinoma tissues were obtained. We identified 2,951 differential genes in the cancer tissue compared to the adjacent tissue. There were 2,532 up-regulated genes and 419 down-regulated genes. Based on three machine learning algorithms, we selected eight signature genes (ENTPD3, PDZD4, CNN1, GTPBP4, FPGS, UTP25, CENPW, and FAM111A). Both internal and external dataset validation and histogram results indicate that these signature genes can accurately predict the progression of gastric cancer. There is limited evidence to support the role of signature genes in gastric cancer. GTPBP4 is highly expressed in gastric cancer tissues, which promotes the progression of gastric cancer progression and may interact with the p53 signaling pathway (21). Low FPGS expression is an independent predictor of poor prognosis in stage II/III gastric cancer patients receiving adjuvant chemotherapy after S-1 surgery (22). However, the relationship between the other 6 characteristic genes and gastric cancer has not been reported.

Cuproptosis is a novel form of programmed cell death associated with copper accumulation, protein lipidation, and mitochondrial respiration (15). Cuproptosis is molecularly distinct from other forms of cell death, such as apoptosis, necrosis, autophagy, and iron death. Copper binding leads to a dangerous increase in lipid-acylated TCA circulating protein function. Excess copper increases lipid-acylated protein aggregation and Fe-S cluster protein instability, leading to protein toxic stress and cell death. As key regulators of cuproptosis, FDX1, and protein-lipid acylation play an important role in this process. Copper ionophores are extremely sensitive to cells that use mitochondrial respiration, which can be explained by their large number of lipid-acylated TCA enzymes. Tumor cells have abnormal mitochondrial metabolism due to the loss of active oncogenes and tumor suppressor genes (23). Aerobic glycolysis is widely observed in activated immune cells in the tumor microenvironment (TME) to support biosynthetic requirements (24). TME is now recognized to play a key role in carcinogenic effects and cancer development. The immune microenvironment is closely linked to the development of tumors (25, 26). It is composed of different types of immune cells and stromal cells that can provide nutritional support to tumor cells. The trace element copper has been reported to play an important role in both cellular and humoral immunity (27, 28), manipulating various immune cells to activate and maintain the immune system (29). In this study, we identified two subtypes C1 and C2 based on cuproptosis. Most immune-related genes were significantly upregulated in the C2 subtype compared to the C1 subtype. We identified C2 as an immune subtype and C1 as a non-immune subtype. The new classification of immune subtypes is helpful for the individualized classification and medication guidance of gastric cancer patients. For small molecule drug screening, we have a list of the top 10 predictors. Numerous studies have confirmed that pemetrexed is a safe and effective drug for the treatment of metastatic gastric cancer (30–32). As a histone deacetylase (HDAC) inhibitor, Vorinostat can be used in combination with capecitabine plus cisplatin (XP) as a therapeutic agent in patients with gastric cancer (33). Dasatinib, which targets a variety of cancer kinases has strong antitumor activity and has been approved for the treatment of leukemia (34). There is increasing evidence that Dasatinib is also effective in gastric cancer (35, 36). Molecular docking showed that Dasatinib could form various forces with CNN1, including hydrogen bonding. The results indicated that candidate drugs may be effective in the treatment of gastric cancer by influencing the expression of characteristic genes. The specific mechanism needs to be further explored. In the future, we plan to establish SD rat gastric cancer model and primary gastric cancer cell model *in vitro*, and use Dasatinib, siRNA and other intervention measures, combined with CCK-8, Western Blot, Scratch assay, immunofluorescence and immunocoprecipitate and other experimental technologies, to explore related molecular mechanisms from multiple perspectives and in all aspects. This will contribute to the development of new targeted therapeutic drugs in molecular pharmacology and help front-line clinical workers to better treat gastric cancer patients. Improve the prognosis of patients, improve life treatment, reduce the burden of family.

This study also has some shortcomings: Firstly, in the gastric cancer samples in the TCGA database selected for this study, the para-carcinoma tissues were not well matched to the cancer tissues, which could lead to false positive results. However, the subsequent validation of external datasets further confirms the reliability of the results. Second, the selected therapeutic drugs in this study were only predicted only by molecular docking without experimental verification. Subsequent *in vivo* and *in vitro* experiments will be carried out to further investigate the relevant molecular mechanisms.

## 5 Conclusion

Eight specific cuproptosis gene diagnostic models and targeted drugs have been identified in gastric cancer, which may contribute to early diagnosis and individualized immunotherapy strategies for gastric cancer patients.

## Data availability statement

The original contributions presented in the study are included in the article/supplementary material. Further inquiries can be directed to the corresponding author.

## Author contributions

LJ conceived and directed the project and wrote the manuscript. JL performed data bioinformatics analyses. YH helped with part of English checking. All authors contributed to the article and approved the submitted version.
